# Effects of Thermal Boundary Resistance on Thermal Management of Gallium-Nitride-Based Semiconductor Devices: A Review

**DOI:** 10.3390/mi14112076

**Published:** 2023-11-08

**Authors:** Tianzhuo Zhan, Mao Xu, Zhi Cao, Chong Zheng, Hiroki Kurita, Fumio Narita, Yen-Ju Wu, Yibin Xu, Haidong Wang, Mengjie Song, Wei Wang, Yanguang Zhou, Xuqing Liu, Yu Shi, Yu Jia, Sujun Guan, Tatsuro Hanajiri, Toru Maekawa, Akitoshi Okino, Takanobu Watanabe

**Affiliations:** 1 Graduate School of Interdisciplinary New Science, Toyo University, 2100 Kujirai, Kawagoe 350-8585, Saitama, Japan; guansujun1222@gmail.com (S.G.); hanajiri@toyo.jp (T.H.); maekawa@toyo.jp (T.M.); 2 Faculty of Science and Engineering, Waseda University, 3-4-1 Ookubo, Shinjuku-ku 169-8555, Tokyo, Japan; caozhi8168@asagi.waseda.jp (Z.C.); c_zheng@aoni.waseda.jp (C.Z.); watanabe-t@waseda.jp (T.W.); 3 School of Engineering, Tokyo Institute of Technology, 4259 Nagatsuta, Midori-ku, Yokohama 226-8502, Tokyo, Japan; xumao@plasma.es.titech.ac.jp (M.X.); aokino@es.titech.ac.jp (A.O.); 4 Graduate School of Environmental Studies, Tohoku University, 6-6-02 Aoba-yama, Sendai 980-8579, Miyagi, Japan; kurita@tohoku.ac.jp (H.K.); narita@material.tohoku.ac.jp (F.N.); 5 National Institute for Materials Science, 1-2-1 Sengen, Tsukuba 305-0047, Ibaraki, Japan; wu.yenju@nims.go.jp (Y.-J.W.); xu.yibin@nims.go.jp (Y.X.); 6 School of Aerospace Engineering, Tsinghua University, Beijing 100084, China; hdwang@mail.tsinghua.edu.cn; 7 School of Mechanical Engineering, Beijing Institute of Technology, Beijing 100081, China; mengjie.song@bit.edu.cn (M.S.); wangw@bit.edu.cn (W.W.); 8 School of Engineering, Hong Kong University of Science and Technology, Clear Water Bay, Kowloon, Hong Kong SAR, China; maeygzhou@ust.hk; 9 Department of Materials, University of Manchester, Manchester M13 9PL, UK; xuqing.liu@manchester.ac.uk; 10 School of Design, University of Leeds, Woodhouse, Leeds LS2 9JT, UK; y.shi2@leeds.ac.uk; 11 School of Engineering and Applied Science, Aston University, Birmingham B4 7ET, UK; y.jia1@aston.ac.uk

**Keywords:** thermal boundary resistance, thermal management, GaN, SiC, diamond

## Abstract

Wide-bandgap gallium nitride (GaN)-based semiconductors offer significant advantages over traditional Si-based semiconductors in terms of high-power and high-frequency operations. As it has superior properties, such as high operating temperatures, high-frequency operation, high breakdown electric field, and enhanced radiation resistance, GaN is applied in various fields, such as power electronic devices, renewable energy systems, light-emitting diodes, and radio frequency (RF) electronic devices. For example, GaN-based high-electron-mobility transistors (HEMTs) are used widely in various applications, such as 5G cellular networks, satellite communication, and radar systems. When a current flows through the transistor channels during operation, the self-heating effect (SHE) deriving from joule heat generation causes a significant increase in the temperature. Increases in the channel temperature reduce the carrier mobility and cause a shift in the threshold voltage, resulting in significant performance degradation. Moreover, temperature increases cause substantial lifetime reductions. Accordingly, GaN-based HEMTs are operated at a low power, although they have demonstrated high RF output power potential. The SHE is expected to be even more important in future advanced technology designs, such as gate-all-around field-effect transistor (GAAFET) and three-dimensional (3D) IC architectures. Materials with high thermal conductivities, such as silicon carbide (SiC) and diamond, are good candidates as substrates for heat dissipation in GaN-based semiconductors. However, the thermal boundary resistance (TBR) of the GaN/substrate interface is a bottleneck for heat dissipation. This bottleneck should be reduced optimally to enable full employment of the high thermal conductivity of the substrates. Here, we comprehensively review the experimental and simulation studies that report TBRs in GaN-on-SiC and GaN-on-diamond devices. The effects of the growth methods, growth conditions, integration methods, and interlayer structures on the TBR are summarized. This study provides guidelines for decreasing the TBR for thermal management in the design and implementation of GaN-based semiconductor devices.

## 1. Introduction

**Figure 1:**
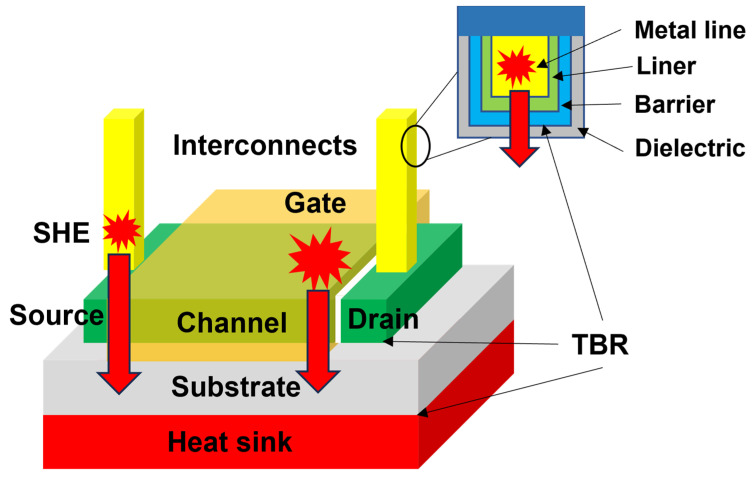
Schematics of SHE in interconnects and transistor channels in semiconductor devices. The joule heat generated in both interconnects and transistor channels passes through several interfaces before dissipating through the heat sink connected to the substrate. The TBR of these interfaces dictates the overall thermal resistance of deeply scaled semiconductor devices.

The self-heating effect (SHE) in semiconductor devices refers to the phenomenon of temperature rise caused by the generation of joule heat as a current flows through transistor channels during operation [1,2,3,4,5]. The channel temperature rise reduces the carrier mobility and the drain current, and it can also induce a shift in the threshold voltage, leading to performance degradation [6]. Furthermore, based on the Arrhenius relationship, which correlates the channel temperature and mean time to failure (MTTF) of a device, an increase in the operating temperature can substantially reduce its lifetime [7,8]. As shown in Figure 1, the current flow through interconnects, which is utilized to connect transistors for power distribution and signal transmission, also causes SHE [9]. Before the 45 nm technology node, the temperature rise within the interconnects was small and negligible. However, aggressive scaling of interconnects notably increased metal resistivity and current density, resulting in a significant increase in temperature owing to the SHE [10,11]. Temperature increase in the interconnects also causes performance degradation and reliability issues owing to electromigration effects. Because of the extremely high thermal resistance of the top printed circuit board (PCB) package layers, the generated heat is dissipated through the heat sink connected to the substrate [10,11,12,13]. Therefore, the thermal conductivity of the substrate materials plays an important role in the heat dissipation of semiconductor devices. The SHE in conventional planar bulk silicon (Si) CMOS devices does not cause a substantial increase in temperature owing to the high thermal conductivity of bulk Si (~150 W/mK). The heat generated in the channel is efficiently dissipated through the Si substrate, preventing a significant temperature increase. Several advanced techniques have been developed to improve the electrical performance of semiconductor devices. For example, silicon-on-insulator (SOI) devices improve performance by reducing the parasitic capacitance. An insulating buried oxide layer, typically SiO_2_, impedes heat dissipation from the channel to the Si substrate because of its low thermal conductivity (~1 W/mK), resulting in severe SHE [3]. Fully depleted silicon-on-insulator (FDSOI) technology has been developed to overcome the short-channel effect. The low in-plane thermal conductivity of ultrathin top Si layers in FDSOI devices also intensifies the SHE [14]. The transition from planar metal-oxide-semiconductor field-effect transistors (MOSFET) to fin-field effect transistor (FinFETs) and gate-all-around field-effect transistor (GAAFET) technologies could significantly improve the performance of transistors. However, in advanced FinFET and GAAFET designs, the SHE becomes more pronounced owing to the encapsulation of the channel by a gate dielectric, with low thermal conductivity coupled with limited heat dissipation paths to the substrate provided by the three-dimensional (3D) structures [15]. Wide-bandgap semiconductors, such as gallium nitride (GaN) and silicon carbide (SiC), offer significant advantages over traditional Si-based semiconductors in terms of high-power and high-frequency operations. The SHE in these semiconductor devices is more severe because of their relatively higher power dissipation. The temperature increase could be as high as 350 °C in the active region of a GaN-on-Si device [16].

The overall thermal resistance of a semiconductor device is the lumped thermal resistance, which comprises the intrinsic thermal resistance of the constituent materials and the thermal boundary resistance (TBR) present at the interfaces between them. At the macroscale, the thermal conductivity of the constituent materials plays a crucial role in determining the heat-dissipation capability of semiconductor devices. However, as technology nodes undergo rapid dimensional scaling, the characteristic lengths of semiconductor devices have reached a point where they are comparable to or even smaller than the mean free paths (MFPs) of heat carriers [17]. For example, atomic layer deposition (ALD) has been employed to fabricate high-k dielectric layers, such as Al_2_O_3_ and HfO_2_, with thicknesses below 10 nm [18]. To reduce the overall electrical resistance of Cu/liner/barrier interconnects, the thickness of liner and barrier layers should be only ~2 nm [19]. These characteristic lengths are considerably smaller than the MFPs of phonons in silicon [20] and even smaller than the MFPs of electrons in silicon and Cu [21,22]. Furthermore, the interface density increases significantly because of the multilevel structure of the interconnect system, becoming even more pronounced in 3D IC architectures [23]. Thermal transport in these nanostructures exhibits a ballistic or quasi-ballistic nature rather than diffusive behavior [24]. Accordingly, the overall thermal resistance of these deeply scaled semiconductor devices is determined primarily by the TBR at the interfaces, rather than by the intrinsic thermal conductivity of the constituent materials. Therefore, the TBR between the different constituent materials should be decreased optimally for adequate thermal management of semiconductor devices.

Here, we provide a comprehensive overview of the effects of the TBR on the thermal management of GaN-based semiconductor devices. This review is organized as follows. Section 2 presents a review of the theoretical and computational methods for TBR prediction and the experimental methods for TBR measurement. In Section 3, recent experimental and simulated TBR data for GaN/SiC and GaN/diamond interfaces are reviewed. The effects of the growth methods, growth conditions, integration methods, and interlayer structures on the TBR of GaN/substrate interfaces are summarized and discussed. In Section 4, the conclusion and an outlook for reducing the TBR for thermal management are provided as a guideline for the design and implementation of GaN-based semiconductor devices.

## 2. Theoretical Methods for TBR Prediction and Experimental Methods for TBR Measurement

### 2.1. Thermal Boundary Resistance

When heat flows across an interface between two dissimilar materials, a temperature discontinuity arises at the interface owing to scattering of heat carriers at the interface. The TBR is defined as the ratio of the temperature difference at an interface to the heat flux flowing across the interface [25,26]. The TBR, also referred to as the interfacial thermal resistance or Kapitza resistance, is the inverse of thermal boundary conductance (TBC). Thermal contact resistance, which occurs at the macroscopic asperity contacts of the surface and air-filled gaps, differs from TBR [27]. In comparison, TBR exists even at atomically perfect interfaces and arises from a mismatch in the energy states of the heat carriers (phonons and electrons) on either side of the interface.

### 2.2. Theoretical and Computational Methods for TBR Prediction

**Figure 2:**
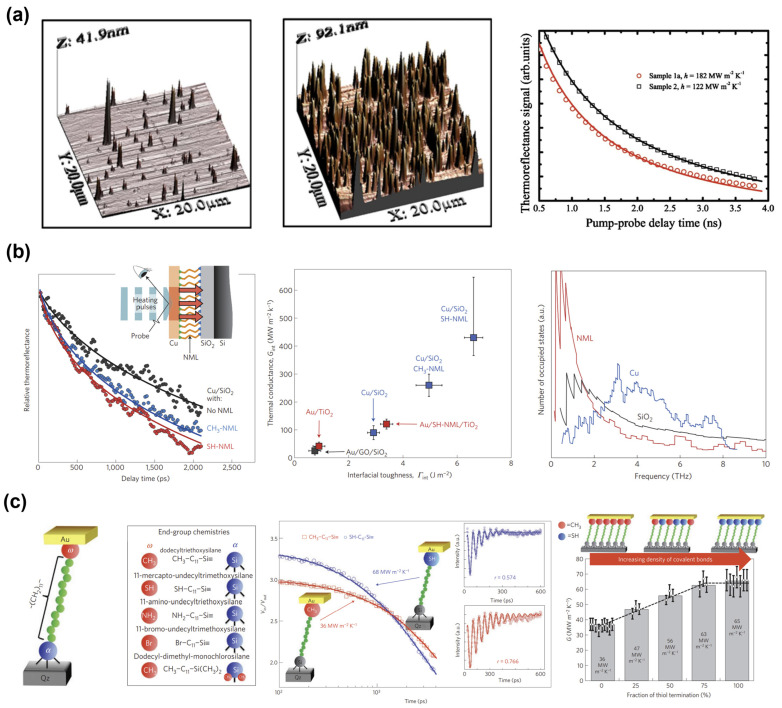
(**a**) Effects of surface roughness on the TBC of Al/Si interfaces. AFM 3D surface profiles for samples 1a (**left**) and 2 (**middle**) prepared using different pre-Al-deposition surface treatments. TDTR data comparison (**right**) shows that the TBC decreases with increasing Si surface roughness (reprinted from Ref. [30]. Reproduced with permission from the American Physical Society (APS). All rights reserved). (**b**) Bonding-induced TBC enhancement using organic nanomolecular monolayer (NML). TDTR data comparison (**left**) for Cu/SiO_2_ interfaces without, and with, a CH_3_- or an SH-terminated NML. The TBC as a function of interfacial toughness (**middle**). Phonon DOS of the Cu, SiO_2_, and SH-NML layers in Cu-NML-SiO_2_ structures produced using MD simulations (**right**) (reprinted from Ref. [39]. Reproduced with permission from Springer Nature publishing. All rights reserved). (**c**) Effects of interfacial termination chemistries on the TBR between a transfer-printed gold film and a self-assembled monolayer (SAM). Depiction of the experimental system consisting of a Qz substrate, bifunctional SAM, and transfer-printed Au layer. List of all SAM chemistries studied and abbreviations used in the text. (**left**). TDTR data comparison for Au/CH_3_–C11–Si≡Qz and Au/SH–C11–Si≡Qz structures (**middle**). The TBC as a function of the methyl:thiol end-group ratio for 0%, 25%, 50%, 75%, and 100% thiol end groups (**right**) (reprinted from Ref. [40]. Reproduced with permission from Springer Nature publishing. All rights reserved).

Various theoretical and computational methods have been employed to predict TBR. The theoretical methods conventionally employed are the acoustic mismatch model (AMM) and the diffuse mismatch model (DMM). In AMM and DMM, the mismatch in the acoustic impedance and phonon density of states (DOS) on both sides of the interface inhibits phonon transmission across the interface [28]. In the AMM, phonons are considered plane waves and the materials through which they propagate are treated as continuous media. In the AMM, a geometrically perfect interface is assumed, and the phonon transport across it is entirely elastic. The transmission probabilities of phonons are determined by calculating the acoustic impedances on both sides of the interface. By assuming the wave nature of phonon transport and specular scattering at the interface, the AMM becomes applicable for predicting the TBR at low temperatures, where long-wavelength phonons dictate the thermal spectrum. In comparison, in the DMM, complete diffuse scattering is assumed to occur at the interface, implying that once a phonon undergoes scattering, it loses all memory of its original direction, polarization, and material of origin, with only its frequency remaining constant. The transmission probabilities of phonons are determined based on the mismatch in the phonon DOS between the two sides of the interface. That is, the transmission probability of phonons with a specific frequency through an interface is significantly higher when matching phonons with the same frequency on the opposite side of the interface. Compared with the AMM, the DMM is more suitable for non-cryogenic temperatures and interfaces with rough surfaces. This suitability is attributed to most acoustic phonons at temperatures of 300 K and above having relatively short wavelengths comparable to the interatomic spacing and surface roughness. In certain studies, instances of agreement have occurred between the TBR values predicted by the AMM or DMM and the experimental results. However, this agreement has been regarded as coincidental because of the simplified assumption regarding the phonon scattering mechanisms occurring at the interfaces [17]. Interfacial properties, such as interfacial disorder, interfacial roughness, interdiffusion, interfacial microstructure, and interfacial bonding [29,30,31,32,33,34,35,36,37,38,39,40], which are not considered in either model, have been shown to have noteworthy effects on the TBR. Therefore, modifications to both models are required to improve their prediction accuracy. For example, Figure 2a shows the effects of surface roughness on the TBR of Al/Si interfaces. Al films were grown on Si substrates subjected to various pre-Al-deposition surface treatments. The measured TBC was found to decrease with increasing Si surface roughness. Figure 2b shows the increase of TBC induced by the bonding enhancement using organic nanomolecular monolayer (NML). The TBC of the Cu/SiO_2_ interface was found to increase with increasing interfacial toughness, which is an indirect measure of bond strength. A fourfold increase in the TBC was observed by using SH-terminated NMLs. The MD simulation results demonstrate that the large NML phonon DOS at low frequencies of <~2 THz form a broad band with an excellent overlap with the SiO_2_ phonon DOS, which increased phonon transmission that leads to a high TBC. Figure 2c shows the effects of chemical bonding on heat transport across interfaces. A gold film was transfer-printed to a self-assembled monolayer (SAM) with systematically varied termination chemistries. The Au/SH–C11–Si≡Qz interface has a higher TBC than the Au/CH_3_–C11–Si≡Qz interface. Furthermore, varying the density of covalent bonds within the SAM modulates both interfacial stiffness and the TBC. The TBC increases with increasing methyl:thiol end-group ratio. More details about the thermal transport in single molecules, single molecule junctions, and self-assembled monolayers are available in some review articles [41,42].

**Figure 3:**
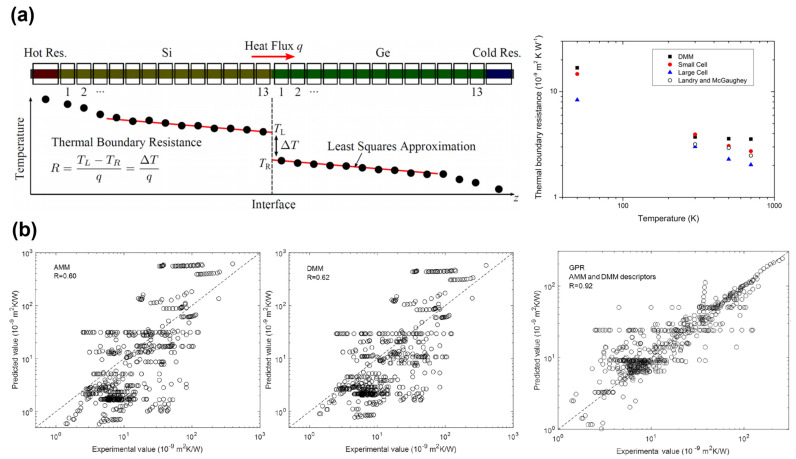
(**a**) Calculation of TBR of Si/Ge interfaces using MD simulations. TBR calculation by least squares linear regression analysis of the temperature profile (**left**). Temperature dependence and size effect of the TBR of Si/Ge interfaces (**right**) (reprinted from Ref. [45]. Reproduced with permission from AIP publishing. All rights reserved). (**b**) Prediction of TBR using machine learning method. Correlation between the experimental values and the predicted values using the AMM (**left**), DMM (**middle**), and machine learning method using Gaussian process regression (**right**) (reprinted from Ref. [48]. Reproduced with permission from Springer Nature publishing. All rights reserved).

Molecular dynamics (MD) simulation is the predominant computational approach for predicting TBR [43,44,45]. Such simulations do not rely on assumptions about the nature of phonon scattering; instead, they require only a description of the atomic interactions as input. However, it remains challenging to establish an appropriate description of atomic interactions that can be applied to diverse systems. Consequently, Si and Ge are frequently chosen as study systems for investigating the TBR in MD simulations. This choice is driven primarily by the availability of reliable interatomic potentials that can accurately replicate the forces experienced by the actual atoms [17]. Figure 3a shows the temperature dependence and size effect of the TBR of Si/Ge interfaces calculated using MD simulations. The MD simulations, limited to the input of atomic interactions, do not allow for the investigation of the contributions of electrons or the effects of electron–phonon coupling on the TBR. In contrast, first-principles calculations can accurately capture the dynamics of various energy carriers, such as electrons, phonons, and magnons, as well as their interactions [46,47]. However, the first-principles calculation method is computationally demanding and time consuming. In recent years, machine learning has emerged as a novel approach for TBR prediction [48,49,50,51,52,53,54,55,56,57], and it has been demonstrated to achieve higher predictive accuracy than the conventionally used AMM and DMM methods [48]. Figure 3b shows the comparison of the correlation between the experimental values and the values predicted by the AMM, DMM, and machine learning method using Gaussian process regression (GPR). However, a lack of available experimental TBR results as training data limits the accuracy of the predictions of this method, as the size of a training dataset is a crucial aspect in determining the quality of the prediction performance in machine learning.

### 2.3. Experimental Methods for TBR Measurement

The following experimental methods are conventionally employed for measuring TBR in thin film samples: the 3*ω* method, Raman thermometry, and transient thermoreflectance (TTR) techniques, including frequency-domain thermoreflectance (FDTR) and time-domain thermoreflectance (TDTR) methods.

**Figure 4:**
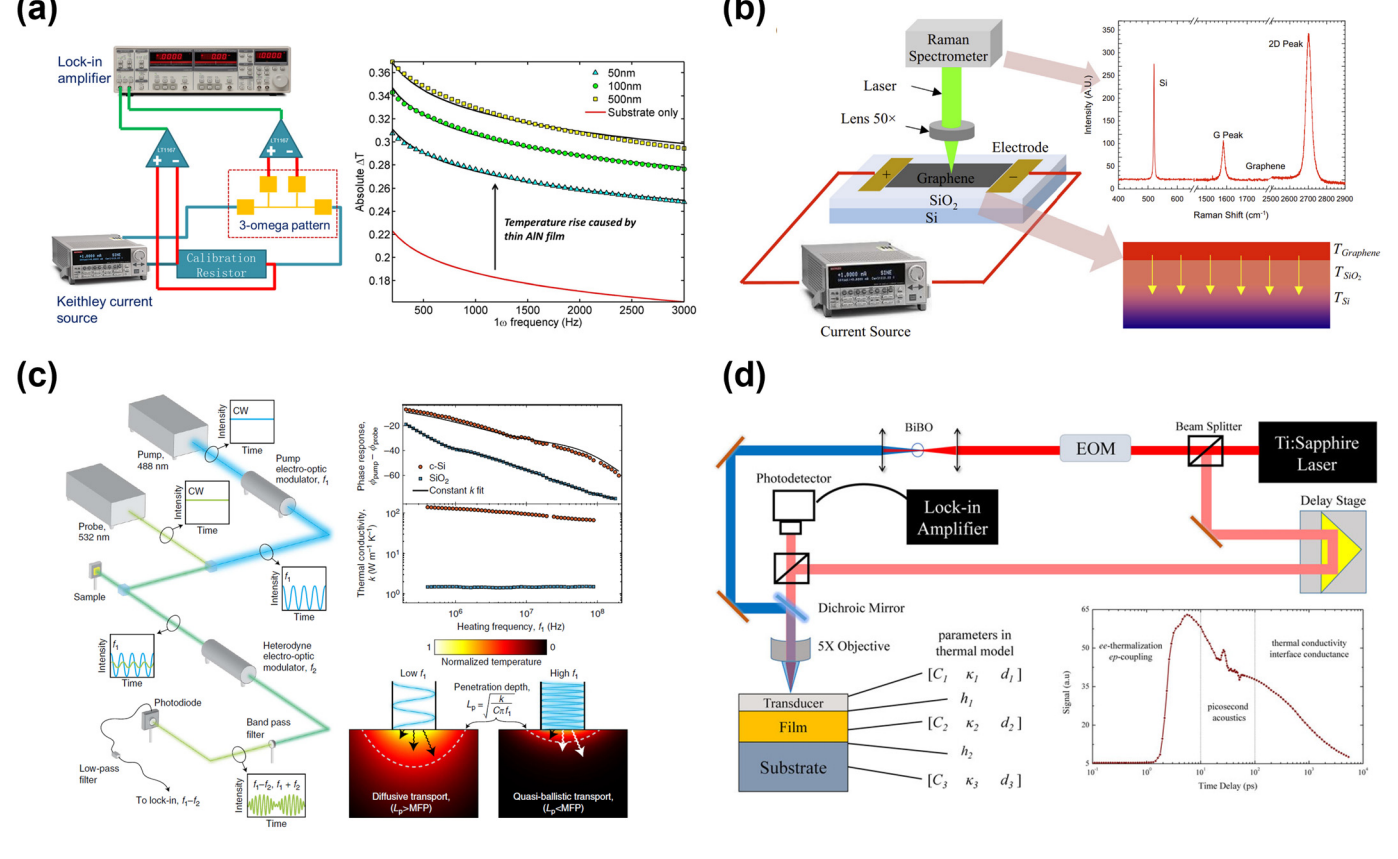
(**a**) Schematic of the experimental setup (**left**) and the raw data (**right**) of the 3*ω* method (reprinted from Ref. [60]. Reproduced with permission from AIP Publishing. All rights reserved). (**b**) Schematic of the experimental setup of the Raman thermometry method (**left**), the Raman peaks (**upper right**), and the temperature distribution (**lower right**) of the Graphene/SiO_2_/Si sample (reprinted from Ref. [61]. Reproduced with permission from Elsevier Ltd. All rights reserved). (**c**) Schematic of the experimental setup (**left**) and raw data (**upper right**) of the FDTR method, and the comparison between diffusive transport and quasi-ballistic transport (**lower right**) (reprinted from Ref. [20]. Reproduced with permission from Springer Nature publishing. All rights reserved). (**d**) Schematic of the experimental setup and the raw data (**lower right**) of the TDTR method (reprinted from ref. [62]. Reproduced with permission from Springer publishing. All rights reserved).

The 3*ω* method is based on the principle of analyzing the temperature-dependent electrical resistance subjected to periodic heating. The TBR can be extracted with known thermal properties adjacent to a thin film of interest. As shown in Figure 4a, a micro-metal strip heater was fabricated on the sample to function as a resistive heater and resistance temperature detector. The heater, driven by an alternating (AC) current with angular frequency *ω*, induces a temperature wave of frequency 2*ω* to diffuse into the substrate. The third harmonic voltage (3*ω*) carries information about thermal transport within the sample. The 3*ω* voltage signal is exceptionally weak and usually approximately three orders of magnitude smaller than the amplitude of the applied 1*ω* voltage, and a lock-in amplifier is usually employed in this measurement technique [58,59].

Figure 4b shows the experimental setup of the Raman thermometry method. The Raman thermometry is based on the principle that the intensity, frequency, and width of Raman peaks are temperature dependent. Among these variations, frequency shift features high sensitivity and is often employed for temperature measurements. When joule heating is used to heat the sample on one side, the temperature of the materials on both sides of the interface can be measured simultaneously by Raman mapping, after which the TBR can be determined from TBR = Δ*T/I^2^R*, where Δ*T*, *I*, and *R* are the temperature difference, applied current, and electrical resistance, respectively [63,64,65].

Both FDTR and TDTR are noncontact optical methods for measuring thermal properties using the thermoreflectance phenomenon. A thin metal layer deposited on top of the sample is used as a transducer, with its reflectance changing with increasing temperature at the laser wavelength. This occurrence facilitates the detection of the thermal response by monitoring the change in reflectance. In FDTR, as shown in Figure 4c, a modulated pump laser beam is incident on the surface of the sample, causing periodic heating and resulting in temperature oscillation. A probe laser beam is used to detect changes in the amplitude and phase of the temperature oscillations as a function of the modulation frequency of the pump beam, after which the TBR is extracted [66,67,68]. A pump–probe configuration is also utilized in TDTR. As demonstrated in Figure 4d, a pump laser beam is incident on the sample surface and heats the sample. After a short delay, the probe laser beam is directed onto the surface of the sample following the pump laser. A detector is used to record the reflected probe beam as a function of time. The TBR can be determined by matching the experimental data with the model predictions [69]. In comparison with the ultrafast lasers employed in TDTR, continuous-wave (CW) lasers used in FDTR offer advantages, such as lower cost and greater wavelength flexibility. However, the FDTR can exhibit higher noise levels. In comparison, the TDTR provides superior signal-to-noise ratios, with ultrafast laser pulses. In addition, the optical alignment process is generally simpler for FDTR, except for extremely high frequencies. However, the TDTR is more sensitive to a wide range of TBRs [70].

## 3. Effects of TBR on Thermal Management in GaN-on-SiC and GaN-on-Diamond Devices

### 3.1. Importance of Thermal Management in GaN-Based Semiconductor Devices

Wide-bandgap semiconductors include various materials, such as GaN, Silicon carbide (SiC), gallium oxide (Ga_2_O_3_), and diamond [71]. Among these semiconductors, GaN has gained significant attention in recent years and, particularly, GaN-based high-electron-mobility transistors (HEMTs) have been used widely for RF frequency applications, such as 5G cellular networks, satellite communication, and radar systems [72].

Initially, AlGaAs/GaAs heterostructures served as the primary channel materials in HEMTs. In recent years, AlGaN/GaN HEMTs have been adopted widely for RF frequency applications. These GaN-based HEMTs have shown significantly higher output powers, thereby surpassing traditional GaAs-based devices by an order of magnitude. Several techniques have been developed to improve the electrical device performance of GaN-based HEMTs, including the mobility, transconductance, cut-off frequency, and output power. However, further improvements have been hindered by significant challenges in thermal management [73]. The performance and reliability of GaN-based HEMTs are limited by the high channel temperature induced by SHE in the device channel [74]. Consequently, to prevent significant thermal damage and ensure long-term reliability, the power dissipation of the current GaN-based HEMTs is reduced for operation at 5–6 W/mm in functional systems, although the theoretical power density can exceed 40 W/mm [75].

The GaN-based HEMTs are conventionally grown on substrates, such as Si, sapphire, and SiC. Si substrates are preferred because of their low-cost mass production. Sapphire substrates are typically employed for GaN-based LEDs. However, the low thermal conductivity of sapphire limits its suitability for high-power applications. SiC substrates are significantly more expensive than Si and sapphire substrates. However, the smaller lattice mismatch (3.8%) and smaller thermal expansion coefficient mismatch (3.2%) between GaN and SiC enable the growth of GaN-based structures with good crystalline quality, making SiC widely used substrates for power electronic applications. The thermal conductivity of the substrate materials is a key factor to consider in the fabrication of GaN-based semiconductors. The thermal conductivity of the substrate material plays a crucial role in limiting the output power densities of GaN-based HEMTs because the joule heat generated by the SHE is primarily dissipated through the substrate. The thermal conductivities of Si, sapphire, and SiC are approximately 130, 30, and 490 W/mK, respectively [76]. Accordingly, SiC substrates are conventionally used for fabricating high-power AlGaN/GaN HEMTs. Currently, GaN-on-SiC devices are the standard for high-power RF applications [77]. Nevertheless, SiC substrates remain inadequate for fully harnessing the total potential of GaN. Among all known natural materials, diamond exhibits the highest thermal conductivity, ranging between 800 and 2000 W/mK, depending on the growth conditions. Diamond has attracted significant interest as a substrate material owing to its exceptional ability to efficiently dissipate heat, surpassing that of SiC [78]. The GaN-on-diamond shows a three-fold increase in the power density and lower junction temperatures compared with that of a GaN-on-SiC device. However, when GaN is integrated with these substrate materials, the presence of a high TBR at the GaN/substrate interface, which could exceed the intrinsic thermal resistance of the substrate materials, could offset the advantages offered by these high-thermal-conductivity substrates. It has been reported that up to 50% of the channel temperature in AlGaN/GaN HEMTs derives from the TBR of GaN/SiC interfaces for high-quality heteroepitaxy [79]. Therefore, several advanced experimental techniques have been developed to lower the TBR and fully exploit the high thermal conductivity of these substrates.

### 3.2. Effects of TBR on Thermal Management in GaN-on-SiC Devices

Gaska et al. first compared the SHE of AlGaN-GaN HFETs grown on sapphire and 6H-SiC substrates. These authors found that heat dissipation strongly affects the device characteristics soon after the application of the source–drain voltage. Thermal resistance is determined primarily by the substrate rather than the AlGaN-GaN active device layer. However, the TBR between the active device layer and substrate was not measured in this study [80].

Kuzmík et al. [81] experimentally investigated and compared the TBR of GaN, Si, SiC, and sapphire substrates. Heterostructures of AlGaN/GaN were grown on different substrates using metal–organic chemical vapor deposition (MOCVD) systems. An AlN nucleation layer of unknown thickness was used. The effective TBR at the GaN/Si interface measured by employing Raman spectroscopy was ~70 m^2^ K/GW, which is in good agreement with the value measured by the transient interferometric mapping (TIM) method [82]. The effective TBR at the GaN/SiC interface was ~120 m^2^ K/GW. However, estimating the TBR at the GaN/sapphire interface is difficult because of the relatively low thermal conductivity of sapphire substrates. The authors assumed that the thermal expansion coefficients, substrate roughness, and defects related to the growth techniques influenced the TBR values. Moreover, they investigated the role of the TBR values for different substrates by calculating the steady-state temperature profiles in the cross-sections of the devices. Using the measured TBR, the highest surface temperature was obtained for GaN grown on sapphire (810 K), and the lowest value was obtained for the SiC substrate (420 K).

Employing Raman spectroscopy, Sarua et al. [83] investigated the TBR of GaN devices grown on various substrates. The effective TBRs of both GaN/Si and GaN/SiC interfaces were determined as ~33 m^2^ K/GW. For the GaN device on the sapphire substrate, the TBR was estimated at approximately 120 m^2^ K/GW. The determined effective TBR is a combined effect of the TBR because of the phonon mismatch and the reduced thermal conductivity of the GaN layer. Furthermore, the effects of the TBR on the temperature increase in ungated AlGaN/GaN field-effect devices were also investigated. The TBRs of the GaN/Si and GaN/SiC interfaces resulted in an increase in the GaN channel temperature by about 30% and 10% for GaN/SiC and GaN/Si devices, respectively, with respect to the case of negligible TBR at the interface. In comparison, despite the high TBR of GaN/sapphire, the influence of TBR on the temperature increase is much smaller at about 2–4%, which is attributed to the low thermal conductivity of the sapphire substrate.

Cho et al. [84] measured the effective TBRs of GaN on Si and SiC substrates with 38 nm and 36 nm thick AlN transition layers, respectively. The measured effective TBRs of the GaN/Si and GaN/SiC interfaces were ~7.8 and ~5.3 m^2^ K/GW, respectively. An approximate solution to the phonon Boltzmann transport equation was used to present a comprehensive model for the effective resistance of the AlN film, indicating that a combination of point defects within the AlN transition layer and near-interfacial defects could be responsible for the effective TBR.

Chen et al. [85] conducted a systematic study on the impact of SiC substrate surface pretreatment on the crystalline quality of subsequently grown AlN nucleation layers and GaN buffer layers in an HEMT structure. These authors also investigated the effects of the structural properties on the TBR using Raman thermography, including the surface of the SiC substrate, as well as the crystalline quality, morphology, and thickness of the AlN nucleation layer. The TBR measurements were performed at an interface temperature of GaN/SiC of approximately 160 °C. Surface pretreatment using H_2_ on the SiC substrate was performed at different temperatures, varying from 1200 to 1280 °C, prior to the growth of GaN/AlN structures. Characterization using X-ray photoelectron spectroscopy (XPS) revealed that the oxygen- and carbon-related contaminants on the SiC substrates were efficiently removed after H_2_ treatment at 1320 °C. The effective TBR was observed to first decrease from ~20 to ~14.5 m^2^ K/GW as the pretreatment temperature increased from 1200 to 1220 °C and, subsequently, it increased to ~33 m^2^ K/GW as the pretreatment temperature reached 1240 °C. The highest TBR value was measured from the sample containing the superior crystalline quality of the AlN nucleation layer in the series, which could be attributed to the degraded surface morphology of the SiC substrates because of the aggressive H_2_ etching process above 1240 °C. This finding revealed the significance of the interfacial structures in the AlN/SiC and AlN/GaN regions in the TBR. Furthermore, by reducing the thickness of the high-quality AlN nucleation layer from 105 to 35 nm, the TBR was reduced from ~33 to ~13 m^2^ K/GW.

Manoi et al. [86] compared the TBR values of various GaN-on-SiC device structures from US, Japanese, and European suppliers, which were all fabricated using standard MOCVD methods. The thickness of the AlN nucleation layers ranged from 40 to 200 nm. Measured using Raman thermography, TBR was found to differ by a factor of four between the different device suppliers, ranging from 15 to 50 m^2^ K/GW at 150 °C. Microstructure/defects within the nucleation layer or near the interfaces hinder heat transport by enhancing the phonon scattering rates, resulting in an increase in the effective TBR. The large variation in the TBR values between different suppliers indicates the potential for nucleation-layer growth optimization using standard MOCVD methods.

Riedel et al. [87] demonstrated that changing over from a MOCVD-grown standard 40 nm thick AlN nucleation layer to a hot-wall MOCVD-grown 80 nm thick AlN nucleation layer could decrease the TBR from ~43 to ~25 m^2^ K/GW at an interface temperature of 100 °C. Such a decrease in TBR leads to a ~10% reduction in the operating temperature of AlGaN/GaN HEMTs. The TEM characterization showed that the AlN nucleation layer, grown using hot-wall MOCVD, was more monocrystalline, thereby resulting in fewer defects and grains and less domain boundary phonon scattering and, consequently, a low TBR.

**Figure 5:**
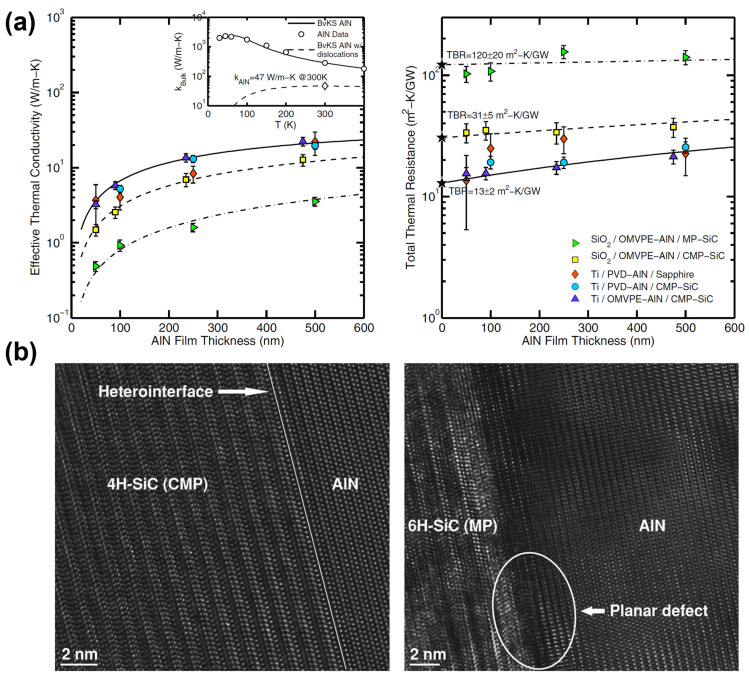
(**a**) Effective thermal conductivity (**left**) and total thermal resistance (**right**) as a function of AlN film thickness on different substrates. These plots indicate that the total thermal resistance mainly derives from the TBR rather than the film itself. (**b**) Comparison of the atomic-resolution TEM images of the interfaces between AlN/CMP SiC and AlN/MP SiC. AlN grown on CMP 4H-SiC substrate has a clearly defined interface with no apparent additional strain in the film (**left**). In contrast, microstructural roughness of the surface of the MP 6H-SiC substrate generates stresses and causes planar defects within the first few atomic layers of the AlN film (**right**). (Reprinted from Ref. [60]. Reproduced with permission from AIP Publishing. All rights reserved).

Su et al. [60] grew AlN thin films on mechanically polished (MP) and chemomechanically polished (CMP) SiC substrates. The TBRs of the AlN/SiC interfaces in the MP and CMP samples were approximately 94 and ~5.1 m^2^ K/GW. The intrinsic thermal conductivities of the AlN thin films for both types of samples showed no remarkable differences at ~47 W/mK. The TBR of ~5.1 m^2^ K/GW is equivalent to ~240 nm of highly dislocated AlN thin films. Figure 5a shows the effective thermal conductivity and total thermal resistance as a function of the AlN film thickness on different substrates. These results indicate that the AlN layer made only a small contribution to the overall thermal resistance. Furthermore, the differences in thermal conductivity between films grown on MP and CMP SiC substrates of differing roughness were found to be more significant than the differences owing to the growth method or substrate material. Atomic force microscopy (AFM) characterization showed that the RMS roughness of MP and CMP substrates are ~1.2 and ~0.2 nm, respectively. Compared with that of the CMP substrate, the RMS roughness of the MP substrate, which is six times greater, is considered responsible for its order-of-magnitude-greater TBR. Atomic-resolution TEM imaging revealed that the near-interface planar defects in the AlN films grown on rough SiC were the source of the increased TBR, as shown in Figure 5b.

Feng et al. [88] reported that a lower TBR of ~20 m^2^ K/GW could be achieved by employing an ultrathin AlGaN buffer layer with a low Al content between the GaN films and SiC substrate in comparison with the TBR of ~25 m^2^ K/GW for the sample with a 90 nm thick conventional AlN buffer layer. An ultrathin AlGaN buffer layer was introduced through trimethylaluminum pretreatment of the SiC substrates. High-resolution XRD and TEM characterization showed that the dislocation density in the GaN layer could be significantly reduced by using an ultrathin AlN buffer layer. The ultrathin buffer layer not only reduces the TBR at the GaN/SiC interface but also improves the crystal quality of the subsequent GaN layer, which is beneficial for improving the device performance.

AlN nucleation layers are conventionally used to improve the wettability of GaN and SiC. Furthermore, AlN layers are expected to serve as phonon bridges to enhance thermal transport between GaN and SiC. However, using an AlN nucleation layer introduces extrinsic thermal resistance owing to the intrinsic thermal resistance of the AlN layer and the TBRs of the GaN/AlN and AlN/SiC interfaces. Therefore, the TBRs of the GaN/SiC interfaces without an AlN nucleation layer were also investigated. Using an RF-plasma-assisted MBE method, Ziade et al. [89] deposited an epitaxial GaN layer directly on a SiC substrate without a transition layer. The TBR of the GaN/SiC interface was measured at ~4.3 m^2^ K/GW using the FDTR method, which was significantly lower than the TBR for a GaN/SiC interface with an AlN transition layer.

Room-temperature surface-activated bonding (SAB) is a promising technique for heterogeneous integration of semiconductor materials and microelectronic packaging. The SAB technique is insensitive to lattice mismatch and can be performed at room temperature and wafer scale, resulting in low thermal stress. Mu et al. [90] directly bonded high-quality GaN to SiC using the room-temperature SAB method. The TBR of the as-bonded GaN/SiC was measured at ~5.9 m^2^ K/GW. The TBR decreased to ~4.3 m^2^ K/GW after annealing at 1273 K for 10 min in flowing N_2_ gas, which is almost the same value as the TBR of directly grown GaN on SiC by MBE. High-resolution scanning TEM and electron energy loss spectroscopy (EELS) were used to study the interface structure and local chemical distribution. The results showed that both the interfacial amorphous layer and interfacial mixing caused by diffusion could have contributed to the low TBR of the as-bonded interface. The decrease in the TBR after annealing is attributed to the disappearance of the amorphous layer and redistribution of Ar atoms. In addition to the decrease in the TBR, the thermal conductivity of the GaN layers is higher than that of the GaN layers grown by MBE, which is beneficial for the heat dissipation of GaN-on-SiC devices.

Theoretical and computational methods have been employed to calculate the TBRs of GaN/SiC interfaces. The TBR at the GaN/SiC interface at room temperature was calculated at ~1.2 m^2^ K/GW using the DMM [91]. In comparison, the calculated TBR of the GaN/SiC interface was indicated as ~2 m^2^ K/GW using nonequilibrium MD simulations [92,93,94]. The measured TBR of GaN/SiC interfaces with different interlayers and different growth or integration methods, and the TBRs predicted using simulation methods are summarized in Table 1.

**Figure 6:**
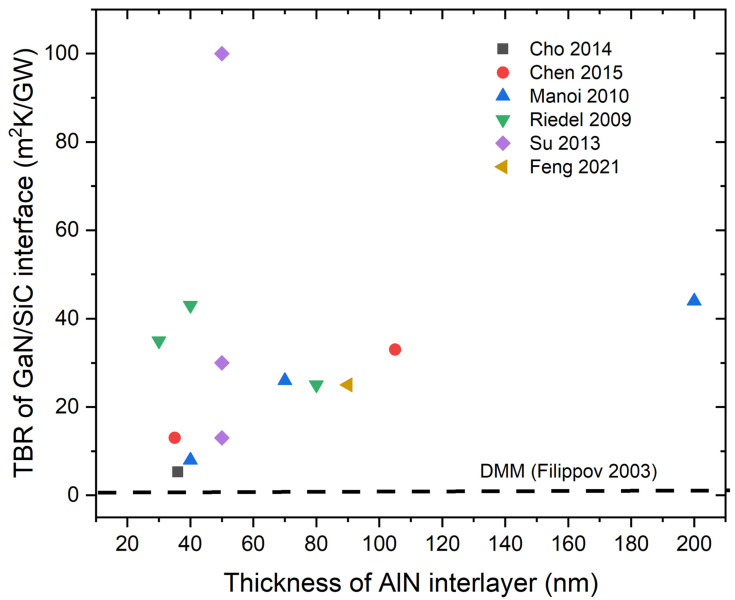
TBR of GaN/SiC interface as a function of AlN interlayer thickness [60,84,85,86,87,88]. TBR predicted by the DMM is also shown for comparison [91]. Only the data with clear interlayer information are included. The TBRs measured by different research groups differ significantly and show no thickness dependence, ranging from 5 to 100 m^2^ K/GW, although the same growth methods are used for growing the AlN nucleation layer.

In summary, SiC substrates are conventionally used for GaN-based HEMTs because of their advantages, which include a small mismatch in the lattice and thermal expansion coefficient with GaN and high thermal conductivity. However, the TBR between the GaN device layer and SiC substrate has a significant effect on the thermal management of GaN-on-SiC devices. The TBRs of the GaN/SiC interfaces were measured as exceeding 100 m^2^ K/GW in some devices, which is two orders of magnitude higher than the values calculated by the DMM and MD simulations (1–2 m^2^ K/GW). The measured TBRs were significantly higher than the intrinsic thermal resistances of the GaN device layer and SiC substrate. An AlN nucleation layer is conventionally used to accommodate the lattice mismatch between the GaN and SiC substrates, resulting in high-quality GaN heteroepitaxy and reduced TBR. However, the TBRs measured by different research groups differed significantly, ranging from 5 to 100 m^2^ K/GW, although the same growing methods were used for the AlN nucleation layers. Furthermore, the TBRs of the GaN/AlN/SiC stacks showed no thickness dependence on the AlN nucleation layer. Some samples with an extremely thin AlN nucleation layer showed a high TBR. Most measured TBRs fell within the range of 10–40 m^2^ K/GW, as shown in Figure 6. This finding indicates that the intrinsic thermal resistance of the AlN layer contributes little to the overall thermal resistance of the GaN/AlN/SiC stacks, which is attributed to the high thermal conductivity of the crystalline AlN nucleation layer. These results demonstrate that the growth method and conditions have a significant impact on the microstructure and defect density near the AlN nucleation layer, resulting in a major difference in the measured TBR. In comparison, GaN/SiC interfaces fabricated by the MBE and room-temperature SAB methods with no AlN interlayer have a much lower TBR, which is close to the TBR values predicted by DMM and MD simulations.

### 3.3. Effects of TBR on Thermal Management in GaN-on-Diamond Devices

Diamond is considered the superior candidate to replace SiC as a substrate for fully exploiting the potential of GaN. Several methods have been employed to integrate GaN with diamond, with the most widely used being the transfer of pregrown GaN from Si to diamond. In this method, the GaN device layer is first grown on a Si substrate, and then polycrystalline diamond is grown by MWCVD on the back of GaN using a transition layer [95]. The second method involves the direct epitaxial growth of GaN on diamond substrates. However, this method is currently uneconomical owing to the unavailability of large diamond substrates [96]. The last method involves direct bonding of diamond wafers to GaN, such as SAB. In this method, the surfaces of diamond and GaN are irradiated simultaneously by an Ar fast atom beam and, after completion of the irradiation process, the diamond and GaN are brought into contact by applying a load for a period [97,98]. In this section, we review the TBRs of various GaN/diamond interfaces integrated using different methods.

Waller et al. [99] prepared a GaN/diamond interface by directly growing diamonds on GaN (van der Waals bonding). The measured TBR of the GaN/diamond interface was approximately 220 m^2^ K/GW, closely matching the value calculated using the weakly bonded AMM model (200 m^2^ K/GW). This value is much higher than that of GaN/diamond with an interlayer. These results indicate that a strong bond is crucial for the successful heterogeneous integration of GaN and diamond, despite the interlayer itself having a lower thermal conductivity and additional extrinsic thermal resistance. A suitable interlayer not only protects the GaN surface during growth but also enables carbide bond formation, which greatly increases the interface adhesion energy and, consequently, facilitates phonon transmission.

To reduce the high TBR of the weakly bonded GaN/diamond interface, an interlayer is required to enhance bonding between GaN and diamond. Numerous research groups have investigated the effects of the interlayer type, including Si, SiC, SiN, and AlN.

Field et al. [100] prepared two GaN-on-diamond samples (namely, one with diamond grown directly on the AlGaN interlayer and the other incorporating a thin crystalline SiC interlayer between AlGaN and diamond). The measured effective TBRs were ~30 and ~107 m^2^ K/GW for the sample with a SiC interlayer and without an interlayer, respectively. The reduced TBR was attributed to the improved adhesion between the SiC and diamond compared with diamond directly on AlGaN because of the increased propensity for carbide bond formation between the SiC and diamond. Stronger carbide bonds aid in the transmission of phonons across the interface, improving heat transport.

Siddique et al. [101] deposited a hot filament (HF) CVD diamond on an AlGaN/GaN HEMT with a 46 nm thick SiN_x_ interlayer. Extremely smooth surface morphology of SiN_x_ was obtained, with an RMS roughness of 0.43 nm. Even with some selective degradation of the in situ SiN_x_ layer, a >20 nm intact SiN_x_ remained that protected the underlying GaN layers. The effective TBR of the GaN/diamond interface measured by TDTR was ~52.8 m^2^ K/GW.

Mandala et al. [102] deposited a thick (>100 μm) diamond layer on 250 nm thick AlN layers. These authors found that a thick diamond layer could not be grown on the untreated as-grown AlN surfaces. However, the successful growth of a thick diamond layer was achieved on AlN surfaces pretreated with 10% N_2_/H_2_ plasma for a minimum of 10 min. The effective TBR of the diamond/AlN interface was measured at approximately 16 m^2^ K/GW. Characterization employing XPS revealed that pretreatment increased the oxygen content on the AlN surface. After pretreatment, O-terminated seeds led to reduced stress at the AlN/diamond interface, resulting in a low TBR.

**Figure 7:**
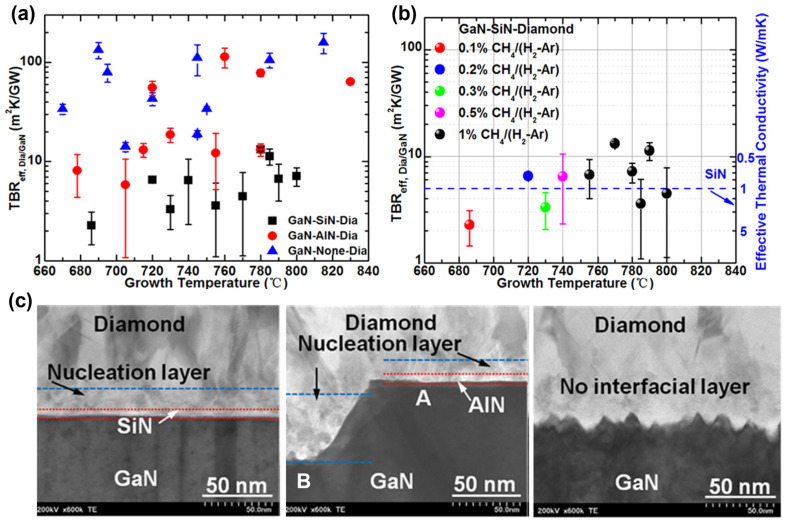
(**a**) Effective TBRs of GaN/diamond interfaces with different interlayers prepared under various diamond growth temperatures. (**b**) Effective TBRs of GaN/SiN/diamond samples as a function of growth temperature for different growth recipes. (**c**) Cross-sectional TEM images of GaN-on-diamond interfaces grown with different interlayers of SiN (**left**), AlN (**middle**), and no interlayer (**right**). (Reprinted from Ref. [103]. Reproduced with permission from ACS Publications. All rights reserved).

Zhou et al. [103] fabricated various GaN/diamond interfaces by growing a polycrystalline diamond layer on a GaN device, with SiN and AlN barrier layers, as well as without any barrier layer, using the MPCVD method. These authors measured and systematically compared the effective TBRs of the GaN/diamond interfaces. The results show that an extremely low TBR of ~6.5 m^2^ K/GW was obtained by using a 5 nm thick SiN barrier layer, whereas the TBR was ~15.9 and ~61.1 m^2^ K/GW for the GaN/diamond interfaces formed by using an AlN barrier layer and without any barrier layer, respectively. In comparison, the DMM-predicted TBR between GaN and diamond was ~3 m^2^ K/GW. Furthermore, no clear correlation was observed between the TBR and diamond growth conditions, such as growth temperature and growth recipes, as shown in Figure 7a,b. The cross-sectional TEM images of the GaN/diamond interfacial region in Figure 7c shows that the low effective TBR of the GaN/SiN/diamond structures could be attributed to the smooth diamond/SiN and SiN/GaN interfaces, leading to low phonon scattering rates. For the GaN/AlN/diamond structures, both the rougher interface and the thicker diamond nucleation layer are responsible for the higher TBR compared with those grown with the SiN barrier layer. When no barrier layer was used, an even larger roughness was observed at the interface between GaN and diamond, resulting in significantly higher TBR.

Yates et al. [104] prepared various GaN/diamond interfaces by growing diamonds on three separate structures (namely, a 5 nm thick SiN interfacial layer, a 5 nm thick AlN interfacial layer, and no interfacial layer). The measured effective TBRs of these GaN/diamond interfaces were ~9.5, ~18.2, and ~41.4 m^2^ K/GW for the SiN and AlN interfacial layers, and no interlayer, respectively. The results showed a trend in TBR difference similar to that measured by Zhou et al. Cross-sectional TEM imaging demonstrated etching of the GaN device layer by the harsh diamond growth environment when an AlN interfacial layer was used, resulting in a rough interface and increased TBR. When no interlayer was used, the diamond was delaminated completely from the GaN layer for most samples, leading to a significantly high TBR. In comparison, high-resolution TEM imaging and EELS analysis revealed that SiN acts as an etch barrier between the diamond and GaN, and a relatively smooth and ordered elemental transition appears throughout the interlayer, thereby reducing disorder and enhancing phonon transport across the GaN/diamond interface.

Huang et al. [105] investigated the effects of interlayer materials on the TBR of a diamond/GaN interface using ab initio calculations incorporating the phonon Boltzmann transport equations. The TBRs of three diamond/GaN interfaces with 5 nm thick Si_3_N_4_, 5 nm thick AlN, and 5 nm thick Si interlayers were calculated at ~4.58, ~5.04, and ~8.48 m^2^ K/GW, respectively. This trend in the TBR difference is in good agreement with the experimental results reported by Zhou et al. and Yates et al. In addition, the effect of the interlayer thickness on the TBR was investigated. The results showed that the optimal interlayer thicknesses were approximately 50–60 nm for the AlN interlayer and 70–80 nm for the Si_3_N_4_ interlayer, matching the spectral phonon transport features. The difference between the experimental and calculated results could be ascribed to the calculations assuming perfectly crystalline interlayer materials without impurities or structural defects. The interlayer materials used in the experiments were either amorphous or contained numerous defects, impurities, and dislocations. In addition, at the nanoscale level, the TBRs measured by different research groups showed substantial measurement uncertainties.

Jia et al. [106] measured the effective TBRs of two types of GaN/diamond interfaces. One had a 100 nm thick SiN interlayer and the other had a 100 nm thick AlN interlayer. The SiN and AlN interlayers were deposited using radio frequency (RF) magnetron sputtering. The TBRs of samples with the SiN and an AlN interlayers were measured at ~38.5 and ~56.4 m^2^ K/GW, respectively, i.e., much higher than the TBRs measured by Zhou et al. and Yates et al. Typically, crystalline SiN and AlN exhibit high thermal conductivities. However, amorphous SiN and AlN thin films deposited by sputtering exhibit much lower thermal conductivities. Therefore, the measured highly effective TBR could be attributed to a thicker interlayer with low thermal conductivity. In addition to the effects of interfacial roughness on the TBR characterized by TEM imaging, the peak shift by XPS analysis demonstrated that compared with AlN, the enhancement in nanodiamond seeding attachment facilitated diamond nucleation and diamond growth on the SiN interlayer, resulting in a lower TBR. Furthermore, interfacial adhesion evaluation using a microscratch test showed that stronger Si–C bonding during diamond nucleation was beneficial for strong film adhesion and lower TBR when SiN was used as an interlayer.

In addition to the effects of the interlayer type, the effects of the SiN interlayer thickness on the TBR have been investigated by several research groups. Sun et al. [107] measured the effective TBRs of a series of GaN/diamond interfaces with SiN_x_ interlayers. Two diamond-growth methods were used (namely, HFCVD and MPCVD). Seventeen wafers were prepared, with a SiN_x_ layer thickness ranging from 28 to 100 nm. These authors found the effective TBR scales with the thickness of the SiN_x_ interlayers. However, no significant differences were observed among the different diamond growth methods. The effective TBR could be reduced from ~50 to ~12 m^2^ K/GW by decreasing the thickness of SiN_x_ from 100 to 28 nm. From the dependence of TBR on the interlayer thickness, the thermal conductivity of the amorphous SiN_x_ layer is estimated at ~1.9 W/mK, in agreement with the values for amorphous silicon nitride thin films. Cho et al. [108] observed reduction by a factor of two in TBR for samples with SiN of different thicknesses. The measured effective TBRs of GaN/diamond interfaces with 22-nm-thick and 31-nm-thick SiN interlayers were ~19.8 and ~31.8 m^2^ K/GW, respectively. Furthermore, these authors calculated the GaN/SiN TBR at ~1 m^2^ K/GW and the SiN/diamond at ~2 m^2^ K/GW using the DMM. Using the calculated DMM value, the intrinsic thermal conductivity of the SiN interlayer was calculated at 1.1–1.5 W/mK. These results show that the low thermal conductivity of the amorphous SiN interlayer is a bottleneck for heat dissipation that needs to be minimized to fully exploit the ultrahigh thermal conductivity of diamond.

Using a combination of Raman thermography and thermal modeling, Pomeroy et al. [109] investigated the role of the diamond substrate thermal conductivity and GaN/diamond TBR in determining the thermal resistance of GaN-on-diamond devices. Two samples were prepared, one with a 95 μm thick HFCVD diamond layer and a 25 nm thick dielectric interlayer and the other with a 120 μm thick MPCVD diamond layer and a 50 nm thick dielectric interlayer. Based on analyses of the lateral and depth temperature profiles, effective substrate thermal conductivities of ~710 and ~1200 W/mK were obtained for the HFCVD and MPCVD polycrystalline diamond wafers, respectively. Effective TBRs of ~27 and ~36 m^2^ K/GW were measured for GaN/diamond interfaces with 25 nm and 50 nm thick proprietary dielectric interlayers, respectively.

Malakoutian et al. [110] deposited polycrystalline diamond on GaN-based HEMTs using MPCVD. By reducing the thickness of the Si_3_N_4_ interlayer to ~1 nm, a record low TBR of ~3.1 m^2^ K/GW was achieved without damaging the electrical performance of the GaN channel, which is close to the values predicted by DMM. However, if the SiN_x_ interlayer is too thin, the harsh H_2_-plasma diamond growth conditions could etch the GaN device layer underneath and degrade its electrical performance. This challenge requires solving.

Jia et al. [111] investigated the effects of a SiN_x_ interlayer structure on the effective TBR of a GaN/diamond interface. Three types of SiN_x_ interlayers were used: 100 nm thick SiN_x_, 80 nm thick SiN_x_, and 100 nm thick SiN_x_ with a 20 nm × 20 nm periodic structure. The effective TBR of the GaN/diamond interfaces was measured with TDTR. The results show that the effective TBR was reduced from ~40.5 to ~38.8 m^2^ K/GW when the thickness of the SiN_x_ interlayer was decreased from 100 to 80 nm. The effective TBR was reduced from ~40.5 to ~32.2 m^2^ K/GW when a 100 nm thick, 20 nm × 20 nm periodically patterned SiN_x_ interlayer was used to replace the 100 nm thick SiN_x_ with no pattern. Imaging employing TEM showed that the periodically patterned structure formed a wavelike SiN_x_/diamond interface, which increased the interfacial contact area and phonon transmission efficiency. In addition, the periodic structure improved the interface bonding strength and seeding density, further enhancing the interfacial heat transfer.

Wang et al. [112] deposited polycrystalline diamonds on GaN using a 30–40 nm thick amorphous SiN_x_ interlayer. At the beginning of diamond nucleation, all samples were pretreated in CH_4_/H_2_ mixtures at positive bias voltage of 400 to 700 V for 60 min at approximately 700 °C. The lowest and highest effective TBRs were measured at ~26 and ~83 m^2^ K/GW for the samples prepared under 700 V and 600 V bias nucleation conditions, respectively. Characterization employing TEM showed that the thicknesses of the SiN_x_ interlayers for the two samples were ~35 and ~70 nm, respectively. Furthermore, Raman spectroscopy showed that different bias voltages led to changes in the interface roughness and microstructure of the transition layer, resulting in differing TBRs.

Sun et al. [113]. fabricated GaN/diamond interfaces using a 40 nm thick amorphous SiN_x_ interlayer. Two samples were prepared: one with a thin diamond nucleation/transition region (<10 nm) at the interface and the other with a thicker nucleation/transition region (estimated 50–100 nm). This variation was controlled using different seeding methods for diamond growth. The former and latter have an effective TBR of ~26 and ~33 m^2^ K/GW, respectively. These results indicate that the nanocrystalline diamond layer near the nucleation surface contributed to the TBR

**Figure 8:**
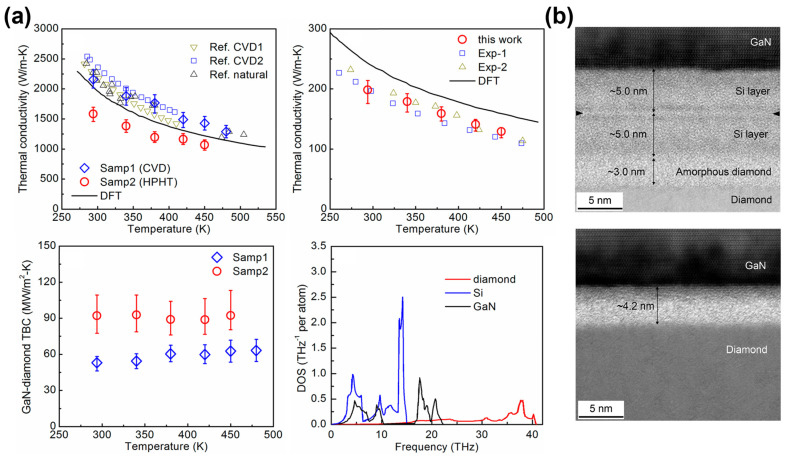
(**a**) Temperature dependence of the measured thermal conductivity of two diamond substrates (**upper left**). Measured thermal conductivity of the GaN layer (**upper right**). Measured TBC of bonded GaN/diamond interfaces (**lower left**), and phonon density of states of GaN, Si, and diamond (**lower right**). (**b**) Cross-sectional HR-STEM images of GaN/diamond interfaces of Samp1 (**upper**) and Samp2 (**lower**). (Reprinted from Ref. [114]. Reproduced with permission from ACS Publications. All rights reserved).

Cheng et al. [114] bonded GaN and a single-crystal diamond using two modified SAB techniques and measured the effective TBR of the GaN/diamond interfaces. The TBR of the first sample with a sputtering-deposited 10 nm thick Si interlayer (Samp1) was ~19.2 m^2^ K/GW. In comparison, a relatively lower TBR of ~10.9 m^2^ K/GW was achieved by mixing Si atoms into the Ar ion beam during SAB processing in the second sample (Samp2), which formed a ~4 nm ultrathin interlayer. Figure 8a shows the temperature dependence of the measured thermal conductivity of the two diamond substrates, the measured thermal conductivity of the GaN layer, the measured TBC of the bonded GaN/diamond interfaces, and the phonon density of states (DOS) of GaN, Si, and diamond. A comparison of the phonon DOS indicates that Si is not an ideal interlayer material from the point of view of phonon DOS mismatch, but it does facilitate strong bonding of GaN with diamond. Therefore, the TBR of bonded GaN/diamond interfaces retains the potential for further reduction using other interfacial layers, such as SiC, AlN, or SiN_x_. The cross-sectional TEM images of the two samples in Figure 8b show that the interlayer thickness in Samp2 was much smaller than that in Samp1, resulting in a relatively lower TBR. Furthermore, device modeling showed that the measured TBR could enable high-power GaN devices by fully exploiting the ultrahigh thermal conductivity of single-crystal diamonds. For the modeled devices, the power density of GaN-on-diamond could reach values ~2.5 times higher than those of GaN-on-SiC and ~5.4 times higher than those of GaN-on-Si with a maximum device temperature of 250 °C.

The TBR has been shown to contribute significantly to the total thermal resistance of GaN-on-diamond devices. Accordingly, the effect of the TBR on the channel temperature rise in the thermal design of GaN-on-diamond HEMTs was investigated. Dumka et al. [115] showed that the effective TBR could be decreased to 18 m^2^ K/GW using a 50 nm thick proprietary transition layer, leading to a more than 25% lower channel temperature rise for GaN-on-diamond HEMTs compared with that of standard GaN-on-SiC HEMTs under a fixed power dissipation condition.

Guo et al. [116] investigated the effects of the TBR of a GaN/diamond interface on the overall thermal management of GaN HEMTs. Employing the finite element method, three-dimensional thermal simulation was performed to analyze the heat dissipation capabilities of different thermal designs. The effect of the TBR on the thermal design of GaN-on-diamond HEMTs, including the GaN buffer, diamond substrates, gate–gate pitch spacing, and chip size, was investigated as the TBR increased from 3 to 140 m^2^ K/GW. The measured TBR of GaN/diamond interfaces with different interlayers and different growth or integration methods, and the TBRs predicted using simulation methods are summarized in Table 2.

Tao et al. [117] performed extensive reverse nonequilibrium molecular dynamics (MD) simulations on a GaN/diamond interface, finding that changing the conventional planar interface to nanoengineered, interlaced architecture with optimal geometry resulted in >80% reduction in TBR. Moreover, introducing a conformal graphene buffer layer further reduced the TBR by ~33%.

Simulation studies demonstrated that in terms of device temperature rise, an HEMT-on-diamond with a GaN/diamond TBR of <30 m^2^ K/GW could outperform an HEMT-on-SiC even with zero GaN/SiC TBR. Therefore, further reduction of the GaN/diamond TBR could enhance the cooling of HEMT-on-diamond devices [73].

**Figure 9:**
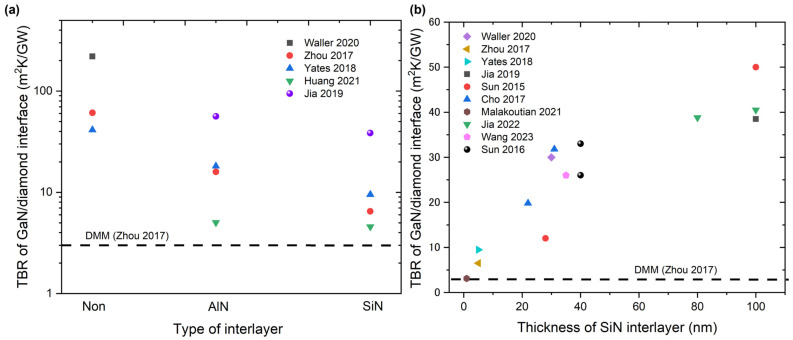
(**a**) Effects of interlayer type on the TBR of GaN/diamond interfaces [99,103,104,105,106]. Using an interlayer decreases the TBR, with SiN being superior to AlN as interlayer in decreasing TBR. (**b**) TBR of GaN/diamond interface as a function of SiN interlayer thickness [99,103,104,106,107,108,110,111,112,113]. TBR predicted by the DMM is shown for comparison [103]. Only the data with clear interlayer information are included. The effective TBR increases with the increasing thickness of the SiN interlayer, which differs from the case of an AlN interlayer in GaN-on-SiC devices, where no thickness dependence could be observed.

In summary, owing to the substantial mismatch in the phonon DOS, a GaN/diamond interface with no interlayer typically has an extremely high TBR, even higher than 200 m^2^ K/GW, which is significantly higher than the intrinsic thermal resistance of GaN active layers and diamond substrates, therefore dictating the total thermal resistance of GaN-on-diamond devices. To reduce the high TBR of GaN/diamond interfaces, interlayers, such as Si, SiC, SiN, and AlN, are typically used to enhance the bonding between GaN and diamond to facilitate phonon transmission, although the interlayer itself, with lower thermal conductivity, introduces additional extrinsic thermal resistance. Figure 9a shows the effects of the interlayer type on the TBR of the GaN/diamond interfaces. The results demonstrate that using an interlayer decreases the TBR and that SiN as an interlayer is superior to AlN for decreasing TBR. This finding is ascribed to SiN being more difficult to etch than AlN in a harsh diamond growth environment, resulting in a smoother interface and a lower phonon scattering rate at the interface. In addition, the stronger Si–C bonds formed between the diamond and SiN facilitate phonon transport across the interface, resulting in a lower TBR. In addition to the experimental results, the ab initio calculation results showed a similar trend in the TBR difference between different interlayers. Furthermore, the effective TBRs increased along with the increasing thickness of the SiN interlayer, as shown in Figure 9b. This finding differs from that for the AlN interlayer in GaN-on-SiC devices, where no thickness dependence could be observed. This could be attributed to the thermal conductivity of the crystalline AlN interlayer, which is one order of magnitude higher than that of the amorphous SiN interlayer. Compared with the effects of defects and disorder near the interface, the intrinsic thermal resistance of the AlN interlayer plays a less important role in the total thermal resistance of the GaN/AlN/SiC stacks. Therefore, optimally reducing the thickness of the SiN interlayer is an effective method to reduce the TBR of GaN/SiN/diamond stacks. Although an extremely low TBR of ~3.1 m^2^ K/GW has been reported, it remains a challenge to reduce the thickness of a SiN interlayer to ~1 nm to reduce the TBR without affecting the electrical performance of GaN active layers. In addition to decreasing the SiN interlayer thickness, introducing nanostructured interfaces, such as periodically patterned SiN interlayers, could increase the effective contact area and improve the interface bonding strength and seeding density, resulting in a low TBR. The calculation results of the MD simulations also showed similar effects of reducing the TBR using nanostructured interfaces. Furthermore, the room-temperature SAB method is promising for decreasing the TBR.

## 4. Conclusions and Outlook

Starting with the SHE-induced temperature rise problem, the effects of TBR on thermal management in GaN-based semiconductor devices were reviewed in this study. The simulation and measurement methods for the TBR were also reviewed. With the rapid dimensional scaling and development of advanced technologies, such as GAAFET and 3D IC architectures, the SHE has become more pronounced and significantly affects the performance and reliability of semiconductor devices. The feature sizes of these semiconductor devices have reached a point where they are comparable with or even smaller than the mean free paths (MFPs) of the heat carriers. Consequently, the overall thermal resistance of these deeply scaled semiconductor devices is primarily determined by TBR. For GaN-based semiconductor devices, such as GaN HEMTs, SiC and diamond are good candidates for thermal management because of their ultrahigh thermal conductivities. However, the presence of a high TBR at GaN/SiC and GaN/diamond interfaces could offset the advantages of their high thermal conductivities. Currently, up to 50% of the channel temperature in GaN-based HEMTs is ascribed to the TBR between GaN and SiC or diamond substrates. Therefore, several advanced techniques have been developed to lower the TBR and fully exploit the high thermal conductivity of SiC and diamond substrates.

Here, we comprehensively summarize the experimental and theoretical TBR values reported in previous studies. For GaN-on-SiC devices, AlN is typically used as a transition layer to relieve stress owing to the large lattice mismatch and thermal expansion coefficients between the GaN layer and SiC substrate. We found that the effective TBRs of GaN/SiC interfaces differed significantly in the range of 5–100 m^2^ K/GW. Furthermore, the TBRs of GaN/SiC interfaces measured by different research groups showed no dependence on the thickness of the AlN transition layer, although the same growth methods were used. This finding indicates that the intrinsic thermal conductivity of the AlN transition layer makes a small contribution to the overall thermal resistance of GaN-on-SiC devices. In comparison, point defects, dislocations, and other disorders introduced by AlN transition layers related to growth techniques significantly affect the TBR values by scattering phonons. Therefore, a thin AlN transition layer with fewer interfacial defects and disorders is the optimal choice for reducing TBR. However, optimizing the growth conditions to minimize the TBR remains a significant challenge. As an alternative to MOCVD, room-temperature SAB is a promising method for reducing TBR by directly bonding SiC and diamond without an interlayer. An extremely low TBR of ~4.3 m^2^ K/GW has been achieved using the SAB method, which is close to the values predicted by DMM. However, even though the TBR of the GaN/SiC interface is reduced to zero, the SiC substrate is less competitive than the diamond substrate with a low TBR in terms of the device temperature rise. Therefore, the TBR of the GaN/diamond interface should be reduced to further unlock the potential of GaN-based semiconductor devices.

For GaN-on-diamond devices, Si, SiC, SiN, and AlN interlayers have been used to enhance bonding between the GaN and diamond substrates to facilitate phonon transport across the interface. The superior candidate for reducing the TBR is SiN because of the smoother interface and stronger Si–C bond formed between diamond and SiN. Differing from the AlN interlayer in the GaN-on-SiC devices, the effective TBRs were found to scale with the thickness of the SiN interlayer in the GaN-on-diamond devices. This finding is attributed to the low thermal conductivity of the amorphous SiN interlayer (~1 W/mK). Therefore, reducing the thickness of the SiN interlayer is an effective method to reduce the TBR of the GaN/diamond interface. However, minimizing the SiN interlayer thickness without affecting the electrical performance of the GaN device layer during the growth of diamond in harsh environments remains a significant challenge. Room-temperature SAB is an alternative method to solve the problem of bonding GaN to diamond. A low TBR of ~10.9 m^2^ K/GW has been achieved by mixing Si atoms into the Ar ion beam during the SAB process, with TBR expected to be further reduced by using other interfacial layers, such as SiC and SiN_x_.

In conclusion, a decrease in the TBR between the GaN and substrate is crucial for thermal management in both GaN-on-SiC and GaN-on-diamond semiconductor devices. Using an interlayer is an effective method to reduce TBR by enhancing the bonding between GaN and the substrate. However, the optimization of the growth method and growth conditions to minimize the defect density near the interface, reduce the phonon scattering rate, and enhance the interfacial bonding to increase the phonon transmission probability across the interface needs further investigation. Furthermore, the SAB method is a promising candidate for replacing the conventionally used CVD growth method. However, the number of studies on this topic is still limited, and further investigation is required.

## Figures and Tables

**Table 1:** TBR of GaN/SiC interfaces with different interlayers and different growth or integration methods. TBRs predicted using simulation methods are also shown for comparison.

Interface	Growth Method	Interlayer	TBR (m^2^ K/GW)	Measurement Method	References
GaN/Si	MOCVD	Unknown	~70	TIM and Raman	Kuzmík [81,82]
GaN/4H-SiC	MOCVD	Unknown	~120	TIM	Kuzmík [81]
GaN/Sapphire	MOCVD	Unknown	Unknown	TIM	Kuzmík [81]
GaN/Si	Unknown	Unknown	~33	Raman	Sarua [83]
GaN/4H-SiC	Unknown	Unknown	~33	Raman	Sarua [83]
GaN/Sapphire	Unknown	Unknown	~120	Raman	Sarua [83]
GaN/Si	MBE	38 nm	~7.8	TDTR	Cho [84]
GaN/4H-SiC	MOCVD	36 nm	~5.3	TDTR	Cho [84]
GaN/4H-SiC	MOCVD	105 nm AlN	~33	Raman	Chen [85]
GaN/4H-SiC	MOCVD	35 nm AlN	~13	Raman	Chen [85]
GaN/4H-SiC	MOCVD	40–200 nm AlN	10–50	Raman	Manoi [86]
GaN/4H-SiC	MOCVD	40 nm AlN	~43	Raman	Riedel [87]
GaN/4H-SiC	MOCVD	30 nm AlN	~35	Raman	Riedel [87]
GaN/6H-SiC	MOCVD	80 nm AlN	~25	Raman	Riedel [87]
GaN/4H-SiC	CMP-SiC	AlN	~5.1	3*ω*	Su [60]
GaN/4H-SiC	MP-SiC	AlN	~94	3*ω*	Su [60]
GaN/4H-SiC	MOCVD	90 nm AlN	~25	TTR	Feng [88]
GaN/4H-SiC	MOCVD	Ultrathin AlGaN	~20	TTR	Feng [88]
GaN/4H-SiC	MBE	None	~4.3	FDTR	Ziade [89]
GaN/4H-SiC	SAB	None	~5.9	TDTR	Mu [90]
GaN/4H-SiC	SAB	None	~4.3	TDTR	Mu [90]
GaN/6H-SiC	Simulation	None	~1	DMM	Filippov [91]
GaN/6H-SiC	Simulation	None	~2.1	MD	Lee [92,93]
GaN/6H-SiC	Simulation	None	~2.4	MD	Hu [94]

**Table 2:** TBR of GaN/diamond interfaces with different interlayers and different growth or integration methods. TBRs predicted using simulation methods are also shown for comparison.

Interface	Growth Method	Interlayer	TBR (m^2^ K/GW)	Measurement Method	Reference
GaN/Diamond	Direct bonding	None	~220	TTR	Waller [99]
AlGaN/Diamond	MPCVD	None	~107	TTR	Field [100]
AlGaN/Diamond	MPCVD	10 nm SiC	~30	TTR	Field [100]
GaN/Diamond	HFCVD	46 nm SiN_x_	~52.8	TDTR	Siddique [101]
AlN/Diamond	MPCVD	None	~16	TTR	Mandala [102]
GaN/Diamond	MPCVD	None	~61.1	TTR	Zhou [103]
GaN/Diamond	MPCVD	5 nm AlN	~15.9	TTR	Zhou [103]
GaN/Diamond	MPCVD	5 nm SiN	~6.5	TTR	Zhou [103]
GaN/Diamond	MPCVD	None	~41.4	TDTR	Yates [104]
GaN/Diamond	MPCVD	5 nm AlN	~18.2	TDTR	Yates [104]
GaN/Diamond	MPCVD	5 nm SiN	~9.5	TDTR	Yates [104]
GaN/Diamond	Simulation	5 nm Si_3_N_4_	~4.58	DFT	Huang [105]
GaN/Diamond	Simulation	5 nm AlN	~5.04	DFT	Huang [105]
GaN/Diamond	Simulation	5 nm Si	~8.48	DFT	Huang [105]
GaN/Diamond	MPCVD	100 nm SiN	~38.5	TDTR	Jia [106]
GaN/Diamond	MPCVD	100 nm AlN	~56.4	TDTR	Jia [106]
GaN/Diamond	MPCVD	28–100 nm SiN_x_	12–50	TTR	Sun [107]
GaN/Diamond	HFCVD	31 nm SiN	~31.8	TDTR	Cho [108]
GaN/Diamond	MPCVD	22 nm SiN	~19.8	TDTR	Cho [108]
GaN/Diamond	CVD	25 nm dielectric	~27	Raman	Pomeroy [109]
GaN/Diamond	CVD	50 nm dielectric	~35.7	Raman	Pomeroy [109]
GaN/Diamond	MPCVD	1 nm Si_3_N_4_	~3.1	TTR	Malakoutian [110]
GaN/Diamond	MPCVD	100 nm SiN_x_	~40.5	TDTR	Jia [111]
GaN/Diamond	MPCVD	100 nm SiN_x_ periodic pattern	~32.2	TDTR	Jia [111]
GaN/Diamond	MPCVD	80 nm SiN_x_	~38.8	TDTR	Jia [111]
GaN/Diamond	MPCVD	70 nm SiN_x_	~83	TTR	Wang [112]
GaN/Diamond	MPCVD	35 nm SiN_x_	~26	TTR	Wang [112]
GaN/Diamond	MPCVD	40 nm SiN_x_	~26	TTR	Sun [113]
GaN/Diamond	MPCVD	40 nm SiN_x_	~33	TTR	Sun [113]
GaN/Diamond	SAB	Si	~19.2	TDTR	Cheng [114]
GaN/Diamond	SAB	Si	~10.9	TDTR	Cheng [114]
GaN/Diamond	CVD	50 nm dielectric	~18	Raman	Dumka [115]
GaN/Diamond	Simulation	None	~3	DMM	Zhou [103]
GaN/Diamond	Simulation	None	16–120	MD	Tao [117]

## Data Availability

Not applicable.
